# White dot syndrome in a patient with presumed ocular tuberculosis: a case report

**DOI:** 10.1186/s40942-022-00372-6

**Published:** 2022-04-05

**Authors:** Zaira Fernanda Martinho Nicolau, Diego Lisboa Araújo, Luis Filipe Nakayama, Vinicius Campos Bergamo, Rodrigo Luz Meirelles, Octaviano Magalhães Júnior

**Affiliations:** Department of Ophthalmology, Universidade Federal de São Paulo-Escola Paulista de Medicina-São Paulo (SP), Botucatu st., 806. Vila Clementino, São Paulo, 04023-062 Brazil

**Keywords:** Multiple evanescent white dot syndrome, Tuberculosis, White dot syndrome

## Abstract

**Background:**

This manuscript describes a case of a patient with presumed ocular tuberculosis masquerading as multiple evanescent white dot syndrome.

**Case presentation:**

A 32-year-old male patient presented with a complaint of reduced visual acuity in the left eye. Retinal fundus exam of the left eye revealed gray-whitish deep lesions predominantly nasal to the optic disc. The lesions were more clearly identifiable on fundus autofluorescence (FAF) imaging, fluorescein angiography (FA) and en face optical coherence tomography (OCT). FA also indicated retinal vasculitis and papillitis. Swept-source OCT B-scan demonstrated loss of the ellipsoid layer in the regions corresponding to the lesions detected by FAF. A positive tuberculin skin test (TST) confirmed presumed tuberculosis, and a related WDS diagnosis was made. Specific antituberculosis therapy was instituted with favorable anatomical recovery and visual outcome.

**Conclusion:**

Multiple evanescent white dot syndrome (MEWDS) may be manifestation of presumed ocular tuberculosis, and multimodal retinal exams can provide a better understanding of atypical diseases and their follow-up.

## Background

White dot syndrome (WDS) is a group of inflammatory ocular disorders manifesting as whitish-yellow lesions affecting the outer retinal layers, retinal pigment epithelium (RPE) and/or choroid. WDS includes acute posterior multifocal placoid pigment epitheliopathy (APMPPE), serpiginous choroiditis, diffuse unilateral subacute neuroretinitis (DUSN), birdshot chorioretinopathy, multiple evanescent white dot syndrome (MEWDS), punctate inner choroidopathy (PIC), multifocal choroiditis with panuveitis, and relentless placoid chorioretinitis. However, overlap of these entities may occur [1].

MEWDS was first reported in 1984 by Jampol as an acute, unilateral and idiopathic retinal disease [2]. MEWDS is usually a self-limited condition with complete recovery of visual acuity. Nevertheless, inflammatory, infectious or neoplastic diseases can mimic or have early manifestations similar to those of MEWDS [3].

This case describes a male patient with presumed ocular tuberculosis masquerading as multiple evanescent white dot syndrome and follow-up with multimodal retinal imaging exams.

## Case presentation

A 32-year-old Caucasian male patient presented with a chief complaint of sudden blurred vision and paracentral visual scotoma in the left eye for the past 15 days. He denied previous similar episodes, ocular pain, flu-like symptoms or other systemic manifestations. Medical and ophthalmological antecedents were unremarkable. Familial history included treatment of his spouse and son for pulmonary tuberculosis in the past year.

Ophthalmological exam disclosed best corrected visual acuity of 20/20 (1.0) in the right eye and 20/25 (0.8) in the left eye. Anterior biomicroscopy was unremarkable, and pupillary exams showed a left relative pupillary defect. Intraocular pressure was 14 mmHg in the right eye and 15 mmHg in the left eye.

**Fig. 1:**
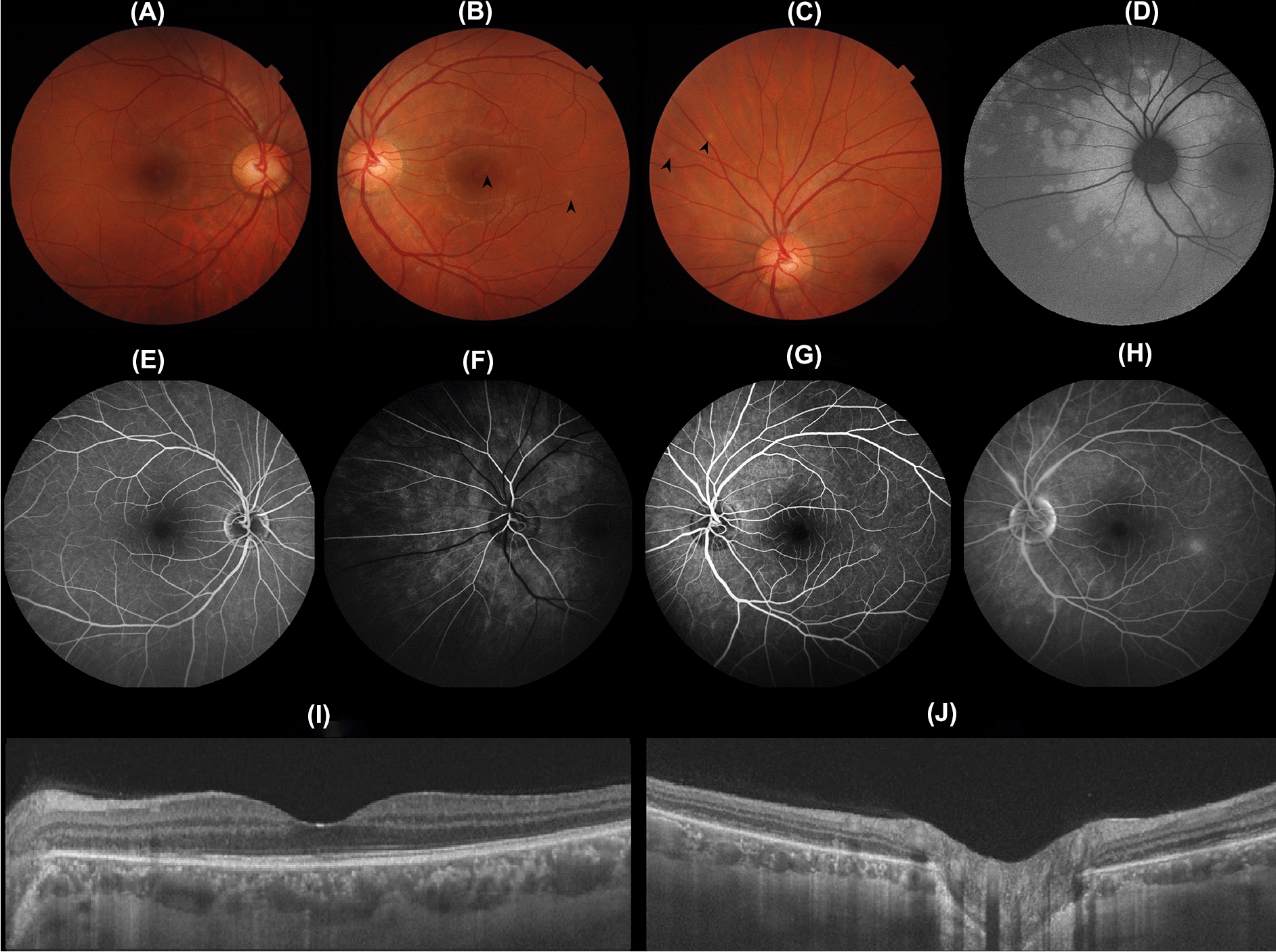
A 32-year-old caucasian male presenting sudden blurred vision in his left eye for 2 weeks. **A** Unremarkable right fundus appearence. **B**, **C** Left fundus showed gray-whitish deep retinal dots (arrow heads). **D** Left fundus autofluorescence (FAF) showing peripapillary hyper-autofluorescent confluent lesions, with adjacent focal circular hyper-autofluorescent lesions. Fluorescein angiography (FA) was unremarkable in the right eye (**E**). Left side showed early and late hyperfluorescent lesions, some of them with wreath-like appearance. Also, a mild disc and peripapillary vessels staining was present (**F–H**). Swept-source optical coherence tomography (OCT) of the left eye revealed focal disruptions of the ellipsoid zone and a choriocapillaris thinning with subjacent pachy vessels at the same locations (**I**, **J**)

Right fundus exam was unremarkable, and left fundus exam revealed a pink optic disc with sharp margins, normal vessels and gray-whitish deep retinal dots predominantly in the upper nasal quadrant (Fig. 1).

As an ancillary exam, fundus autofluorescence (FAF) showed peripapillary hyper-autofluorescent confluent lesions with adjacent focal circular hyper-autofluorescent lesions in the left eye (Fig. 1). Fluorescein angiography (FA) showed early and late hyperfluorescence in the same regions as the lesions detected by FAF, and some of the lesions had a wreath-like appearance. Mild disc staining was also present, just like the peripapillary vessels (Fig. 1).

Swept-source optical coherence tomography (OCT) of the left eye revealed focal disruptions of the ellipsoid zone in the areas corresponding to the lesions seen on FAF. Choriocapillaris thinning with subadjacent pachy vessels was observed at the same locations (Fig. 1). FAF, AF and OCT of the right eye were unremarkable. OCT-angiography (OCTA) showed normal vascular structures in the superficial, deep and choriocapillaris layers of both eyes. En face OCT showed normal superficial retina, deep retina, outer retina and choriocapillaris in both eyes. Manual correction of automatic segmentation of retinal layers was performed to avoid segmentation artifacts. In this case, en face OCT did not reveal hemorrhages or opacities, which may lead to shadowing and projection artifacts.

Laboratory workup included normal complete blood count, erythrocyte sedimentation rate of 3 mm (upper threshold of 15 mm), negative treponemal and nontreponemal syphilis tests, negative IgG and IgM toxoplasmosis, and negative hepatitis B, hepatitis C and human immunodeficiency virus (HIV) serologies.

However, a tuberculin skin test (TST) was positive with a 19-mm induration. Chest radiography and pulmonary workup were unremarkable. Cranial contrast-enhanced computed tomography (CT) showed increased thickness of the left intraorbital optic nerve with late contrast enhancement.

**Fig. 2:**
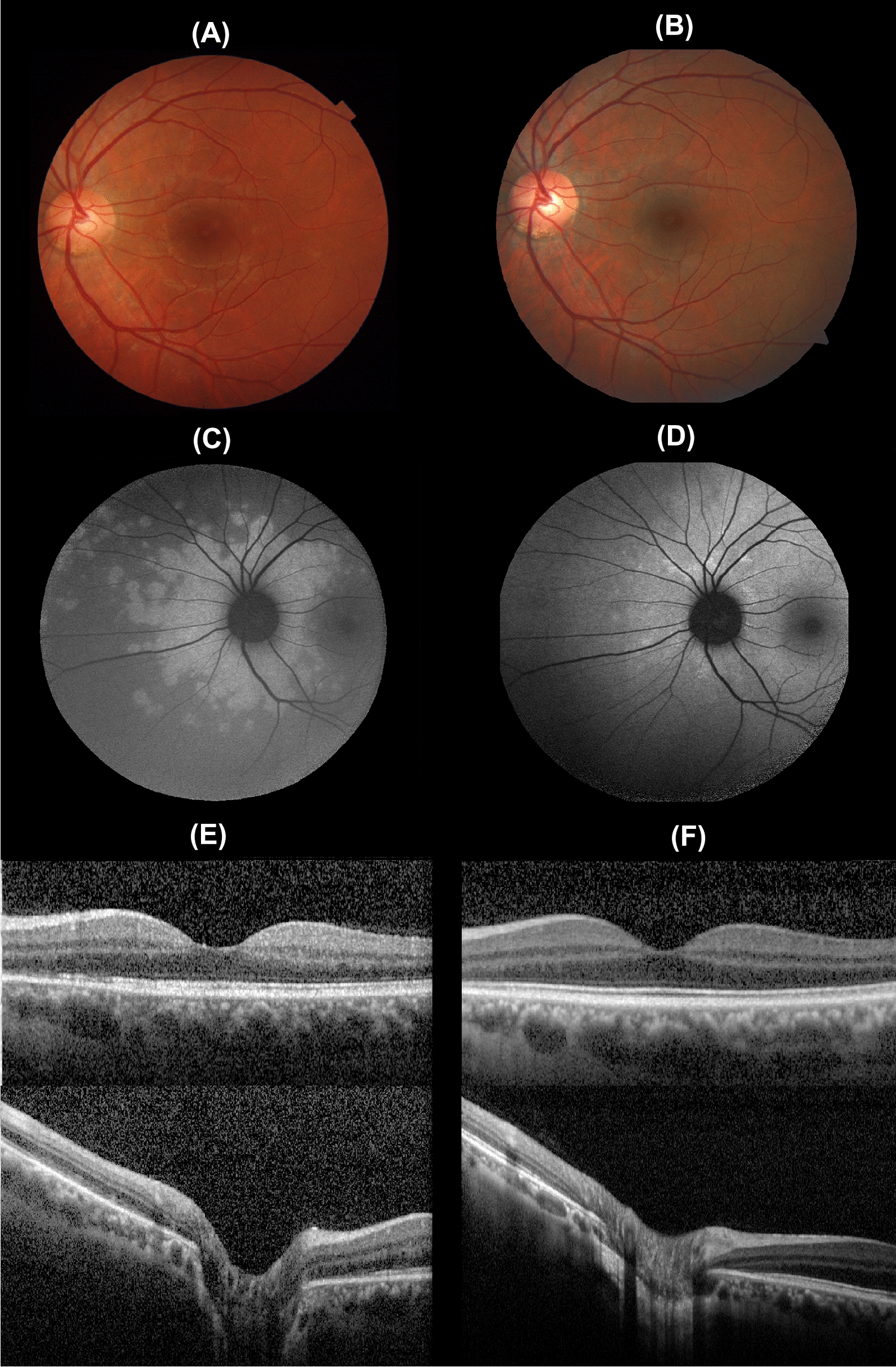
Five months after the treatment the white dots could not be seen on fundus examination (**B**). **D** Improvement of peripapillary lesions on FAF. **F** Disruptions of the ellipsoid zone also showed improvement on OCT. Exams at presentation (**A**, **C**, **E**)

The patient received treatment for presumed tuberculosis with systemic rifampin, isoniazid, pyrazinamide and ethambutol for 2 months, followed by 4 months on rifampin and isoniazid only. Five months after treatment, the patient presented improvement of visual acuity in both eyes (20/20), with no white dots on fundus examination, and FAF and FA exams were also improved (Fig. 2).

## Discussion and conclusions

MEWDS is an acute and typically unilateral retinal disease that predominantly affects young females; its exact pathogenesis is unknown. Findings of MEWDS include nummular white lesions, macular granularity, optic disc edema, enlargement of the blind spot and relative afferent pupillary defect [2, 4, 5].

OCT in MEWDS demonstrates disruption of the outer segments, irregularities of the RPE and confluent spots or plaque on the outer retina. Therefore, MEWDS is considered a disease of the photoreceptors [6]. FAF reveals hyper-autofluorescence areas that correlate with those with early hyperfluorescence in FA [6]. A possible explanation for these findings is increased inflammation extending to deep retinal capillaries or activation of microglial cells [7].

In this case report, the patient presented white-circular outer retinal lesions associated with a hyper-autofluorescence pattern, early hyperfluorescence on FA and disruption of the outer segments on OCT, which are features compatible with MEWDS. However, he also had atypical features, such as male gender, history of family members with past tuberculosis, and vasculitis seen on FA. The presence of atypical features such as older onset age, past medical history of malignancy or immunosuppression, bilaterality, pronounced ocular inflammation, and lack of spontaneous improvement demands additional investigation [3]. It should be considered inflammatory, infectious, and neoplastic diseases, such as sarcoidosis, syphilis, tuberculosis, and primary vitreous lymphoma, among others [3].

TST positivity does not distinguish between latent or active tuberculosis, but an induration of 15 mm or more is considered positive even with no risk factors for tuberculosis [8]. A positive TST test should be followed by symptoms assessment, physical exam, and chest radiograph. The treatment for latent TB (LTBI) should be encouraged to reduce the risk of progression to active TB. For instance, 80% of the US TB cases are from progression of untreated LTBI [8]. The possibility of ocular tuberculosis should be investigated, especially in patients with epidemiological history, vitreous opacification, optic neuritis, vasculitis, and choroidal tubercles [9]. Tuberculosis-related WDS presents most commonly as serpiginous-like choroiditis. This entity typically manifests as multifocal lesions that are noncontiguous with the optic disc, serpiginous spread and stippled hypo and hyper autofluorescence [10]. Choroidal tubercles are another common form of ocular tuberculosis and appear as white-yellowish lesions, usually in the posterior pole and with early hypofluorescence and late hyperfluorescence [3]. Tuberculosis can also manifest as APMPPE but with characteristics different from those encountered in our patient. This form of tuberculosis usually appears as a yellow-white plaque on fundus examination, with early hypofluorescence and late staining on FA, elevations of the ellipsoid zone and disorganization of outer retinal layers on OCT, and capillaris hypoperfusion on OCTA [10].

This case reports a patient with MEWDS-like features with presumed ocular tuberculosis and his improvement on ophthalmological and retinal ancillary exams after tuberculosis treatment. The presence of atypical WDS findings in patients should raise the possibility of undiagnosed inflammatory, infectious or neoplastic disease. Multimodal retinal exams enable a better understanding of atypical diseases and their follow-up. The knowledge of these entities is important for proper diagnosis, management, and favorable final visual outcome.

## Data Availability

All data analyzed during this study are included in this published article.

## Abbreviations

WDS: White dot syndrome
RPE: Retinal pigment epithelium
APMPPE: Placoid pigment epitheliopathy
DUSN: Diffuse unilateral subacute neuroretinitis
MEWDS: Multiple evanescent white dot syndrome
PIC: Punctate inner choroidopathy
FAF: Autofluorescence
FA: Fluorescein angiography
OCT: Optical coherence tomography
TST: Tuberculin skin test
CT: Computed tomography
HIV: Human immunodeficiency virus
